# Change in lifestyle and mental health in young adults: an exploratory study with hybrid machine learning

**DOI:** 10.3389/fpubh.2025.1562280

**Published:** 2025-06-04

**Authors:** Nayoung Park, Hyekyung Woo

**Affiliations:** Department of Health Administration, Kongju National University, Gongju, Republic of Korea

**Keywords:** young adult, mental health, depression, subjective stress, machine learning

## Abstract

Various mental disorders are becoming increasingly prevalent worldwide. Young adults are particularly vulnerable to mental health issues amid rapid lifestyle changes and socioeconomic pressures. This study adopted hybrid machine learning methods, combining existing statistical analysis and machine learning, to determine which factors affect young adults’ mental health, considering recent changes. We used 4-year data (2019–2022) derived from the Community Health Survey, and the final study sample included 141,322 young people aged 19–34. We selected variables based on a literature review and feature selection and performed complex sample logistic regression analysis. New variables that had not previously been discussed (unmet medical needs, chewing difficulty, and accident/addiction experiences) were derived and found to significantly impact depression and subjective stress. These factors’ impact on mental health was generally greater than that of the theoretical background variables. In conclusion, this study emphasizes the need to consistently monitor various factors in today’s rapidly changing environment when devising policies aimed at managing young adults’ mental health.

## 1 Introduction

Various mental disorders are continuously becoming increasingly prevalent worldwide (1), with young adults among society’s most vulnerable to mental health disturbances, and the general health loss associated with these issues peaks among people aged 25–35 (2, 3). Young adults transitioning from adolescence to adulthood are also considered particularly vulnerable to depression and stress (4). Mental health exhibits different patterns according age (5), and the incidence of chronic problems, including anxiety disorders and depression, increases during young adulthood (6). Mental health problems developed in adolescence often persist in young adults (7), and these problems may be exacerbated by comorbid mental disorders, such as functional impairment as a consequence of depression (8).

It is crucial to appreciate the factors that can cause mental health to deteriorate differentially for each age group. Young adults are widely exposed to psycho-emotional difficulties associated with life changes and pressures from academic study, employment, marriage, childbirth, and financial independence (9). Compared to other age groups, young adults who experience poor mental health are more likely to suffer from long-term mental disorders (10). While children and adolescents are typically supported and guided by parents and school systems (11) and relatively stable social safety nets are in place for those in middle and old age (12, 13), young adults are highly susceptible to mental health difficulties owing to the pressures of newfound independence and social instability.

The factors that affect young adults’ mental health may be personal, socioeconomic, environmental, or cultural. Previous studies have examined the influences of lifestyle (14), physical activity (15), employment (16), academic study (17), family environment (18), housing type (19), and stigma (20). These factors are major risk factors in young adults’ adverse mental health (21, 22). However, socioeconomic and cultural environments have recently changed at an unprecedentedly rapid pace, including changes in educational environments and occupational structure (23) and improved access to digital environments (24), significantly influencing the lifestyles and structures of young adults (25). To reflect these rapid environmental changes, it is important to continuously monitor the risk factors for mental health disturbances within wider theoretical considerations.

Recently, there have been attempts to use artificial intelligence (AI), such as machine learning (ML) and deep learning, to study this topic (26–28). To reflect the changing environment, it is necessary to accumulate quantitative and qualitative results for each life stage using the latest data. ML techniques are practical for handling vast amounts of unstructured data (29), which makes it useful for identifying risk factors that are difficult to detect with traditional statistical methods. However, ML has limitations with respect to interpreting relationships between variables (30). One effective means of addressing these limitations is to combine ML techniques with traditional statistical analysis methods.

This study explored the factors affecting the mental health of young adults using a hybrid machine learning/traditional statistics technique. Specifically, we confirmed theoretical variables based on a literature review, selected variables via ML, compared and evaluated models, and confirmed the associations between the new variables selected by ML. Our method can flexibly explore the factors that influence young adults’ mental health in response to environmental changes.

## 2 Methods

### 2.1 Data resources and participants

We used data from the Community Health Survey (CHS) organized by the Korea Disease Control and Prevention Agency. The CHS is a nationwide and representative survey that has been conducted among adults aged 19 or older every year since 2008. The survey includes various health-related questions, covering health behaviors, mental health, unmet medical needs, and social and physical environments. The sampling process involves stratification by housing type (Dong/Eup/Myeon), followed by a two-stage sampling method. First, the sample regions are selected via probability proportional systematic extraction; then final households are selected through systematic extraction. The CHS operates a 4-year survey cycle, and we used data from the period 2019–2022. From among the survey’s 919,395 participants, 141,322 young people aged 19–34 were ultimately selected after excluding missing data and incomplete responses. This study was granted exemption by the Institutional Review Board of Kongju National University in view of using raw data from the Community Health Survey in Korea (IRB number: 2023-102).

### 2.2 Study design

**Figure 1:**
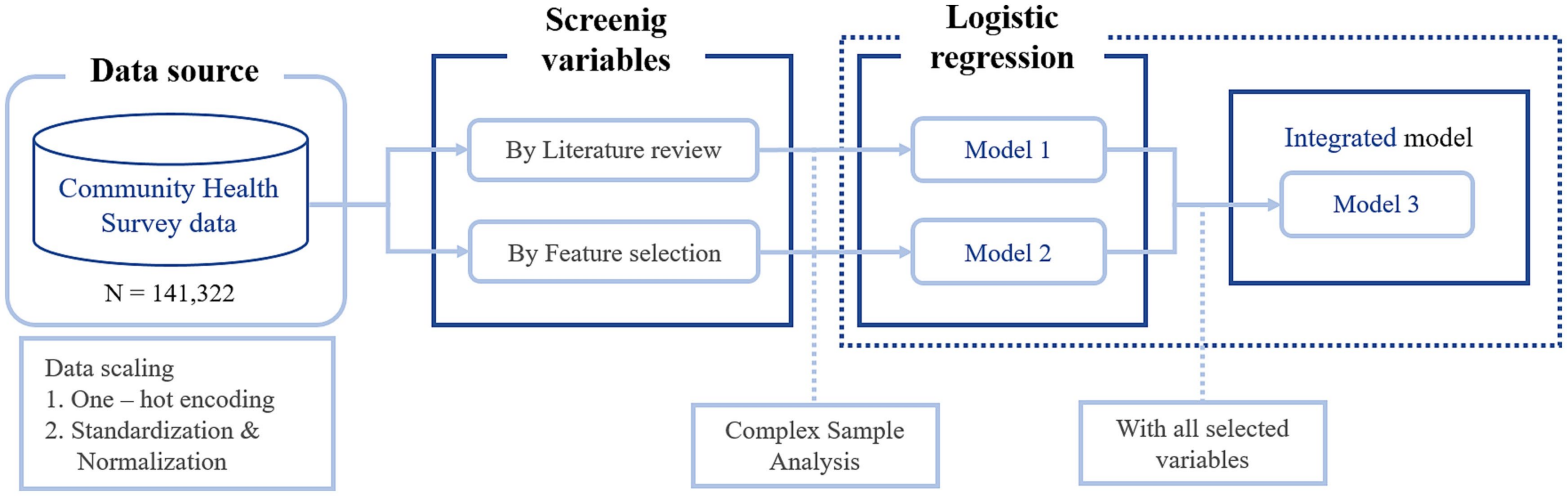
Flow chart of the study.

We used a hybrid machine learning approach that combines traditional statistical analysis with ML. Two main processes were used to select the factors influencing young adults’ mental health. First, we searched the literature, selecting 15 papers from PubMed published within the last 10 years. The following search string was used: (mental health OR depression OR depressive OR stress) AND (young adult OR young people). We also selected 15 Korean papers from Google Scholar because we used survey data from the Korean context. Next, variables were selected by feature selection on all variables in the data. Feature selection can reduce model complexity and improve performance by removing irrelevant variables to identify the optimal subset from a wide range of data (31). Following each process, we constructed Models 1 and 2 and performed logistic regression analysis to evaluate the impact of the selected variables on the mental health of young adults. Finally, we compared the impact of variables in an integrated model (Model 3) based on all selected variables. The overall flow of the study design is presented in Figure 1.

### 2.3 Variables

Mental health-related variables were defined as the experience of depression and subjective stress level. Depression experience was assessed using the CHS item, ‘During the past year, have you ever felt so sad or hopeless for two consecutive weeks or more that it disrupted your daily life?’ Respondents who answered ‘yes’ were classified as having experienced depression. Subjective stress level was measured by asking, ‘How much stress do you usually feel in your daily life?’ Those who answered ‘very much’ or ‘a lot’ were classified as experiencing a high level of subjective stress. For logistic regression analysis, demographic variables such as sex, age, education level, marital status, economic activity, and residential area were designated as basic adjustment variables. Detailed variables from the CHS used in the study, including demographic, behavioral, and health-related categories such as smoking, drinking, physical activity, and mental health items, are presented in Table 1.

**Table 1:** Categories and items of the Community Health Survey.

Category	Survey item
Household	Household type, Status as a recipient of basic livelihood support, Household income
Smoking	Current smoking
Drinking	Lifelong drinking
Safety sense	Wearing seatbelts
Physical activity	Intense to moderate physical activity, Walking, Metabolic equivalent of task
Dietary life	Eating breakfast, Reading nutrition labels
Oral health	Subjective oral health level, Chewing difficulty
Mental health	Sleep, Stress, Depressive experience
Disease	Recognition of early symptoms of stroke/myocardial infarction, Hypertension, Diabetes
Medical Use	Unmet medical needs
Accident/Addiction	Accident and addiction experience
Quality of life	Subjective health level

### 2.4 Data analysis

We analyzed the data using R version 4.3.1 and IBM SPSS Statistics 25.0, with a significance level set at *α* = 0.05. Feature selection was conducted using lasso regression to identify factors influencing mental health. Lasso is an extension of generalized linear regression, which continuously performs shrinkage operations to reduce the possibility of model overfitting (32). It reduces the variance of regression coefficients and selects more relevant and interpretable variables from large sets of multicollinear variables (33). Considering that the CHS has a complex sample design, we performed complex sample logistic regression considering weights.

## 3 Results

### 3.1 Screening factors affecting mental health

**Figure 2:**
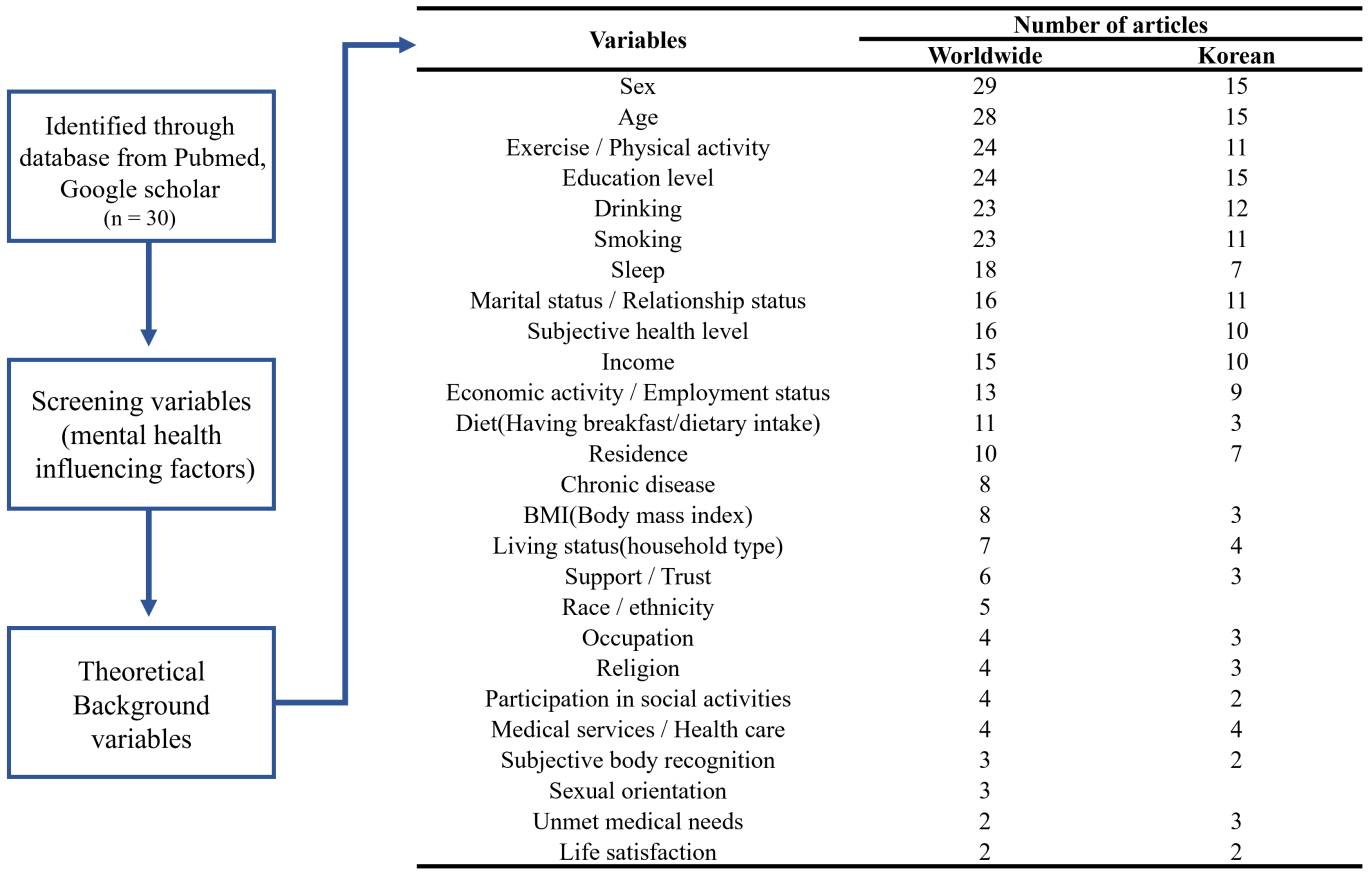
Flow chart of the literature review.

**Figure 3:**
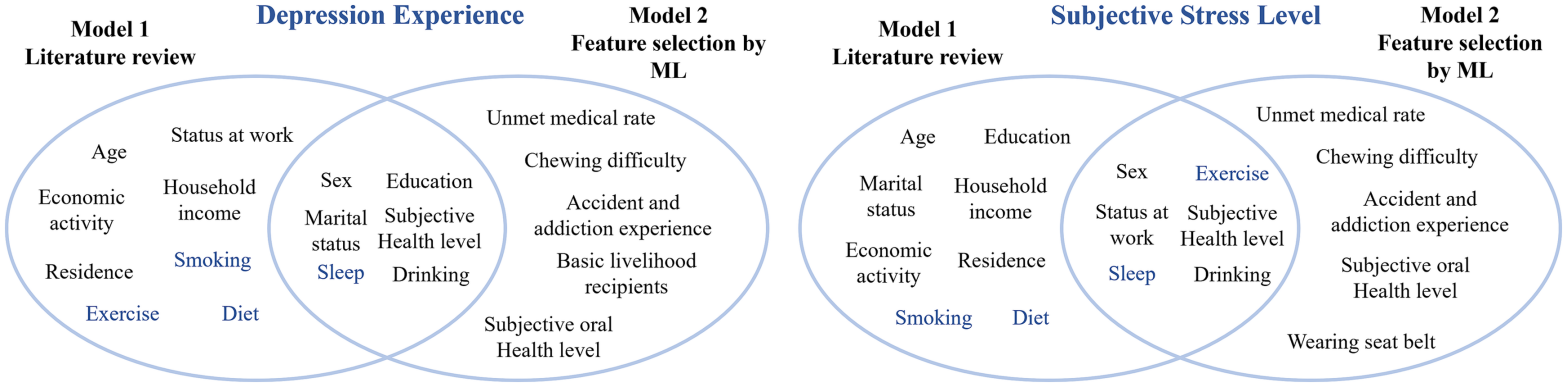
Diagram of selected variables.

Figure 2 presents the outcome of the literature review, including the screening steps and the distribution of mental health-related variables identified from both global and Korean studies. Subjective health level, smoking, drinking, breakfast consumption, sleep duration, and exercise were selected as theoretical background variables, excluding sociodemographic variables. As a result of feature selection for mental health variables, subjective health level, sex, sleep, and drinking were commonly selected among the theoretical background variables. Excluding variables overlapping with the theoretical background, unmet medical needs, chewing difficulty, the number of accidents and addiction experiences, and subjective oral health were selected. Figure 3 presents the variables selected based on the literature review and feature selection. In this study, variables that selected only through feature selection were designated key factors, using the top 3–5% as a cutoff.

### 3.2 Complex sample logistic regression analysis

Tables 2, 3 present the results of a complex sample logistic regression analysis that adjusted for age, sex, education, marriage, economic activity, and region to investigate the impact of selected independent variables on mental health. All variables in Models 1 and 2 had a statistically significant impact on depression (*p* < 0.001). In the theoretical background variables, subjective health showed the strongest association, indicating that worse subjective health increased the likelihood of experiencing depression. For key factors, people experiencing unmet medical needs or discomfort when chewing showed a greater tendency to experience depression. A higher number of accident/addiction experiences also increased the likelihood of experiencing depression.

**Table 2:** Complex sample logistic regression results (depression experience).

Variables			Depression experience (ref: no)				
			Model 1			Model 2	
			OR(p)	95% CI		OR(p)	95% CI
Literature review	Subjective health level	Good	1.00			1.00	
		General	2.11^*^	(2.07–2.14)		1.96^*^	(1.93–1.99)
		Bad	6.22^*^	(6.01–6.36)		5.00^*^	(4.88–5.12)
	Smoking	No	1.00				
		Yes	1.71^*^	(1.68–1.75)			
	Drinking	No	1.00				
		Yes	1.19^*^	(1.16–1.22)			
	Having	Yes	1.00				
	Breakfast	No	0.98^*^	(0.97–0.99)			
	Sleep	≥7 h	1.00				
		<7 h	1.36^*^	(1.34–1.38)			
	MET	<600	1.00				
		≥600	1.26^*^	(1.24–1.28)			
Feature selection	Unmet medical needs	No				1.00	
		Yes				2.43^*^	(2.37–2.50)
		Never needed				0.89^*^	(0.87–0.91)
	Chewing difficulty	No				1.00	
		General				1.55^*^	(1.51–1.58)
		Yes				2.01^*^	(1.95–2.06)
	Accident and addiction experience	No				1.00	
		1–2				1.85^*^	(1.79–1.90)
		More than 3				3.15^*^	(2.79–3.56)
Cox and Snell *R*^2^			0.036			0.041	
Nagelkerke *R*^2^			0.098			0.110	

Adjusted for age, sex, education, marriage, economic activity, and region.

Model 1: theoretical background variables identified in a literature review; Model 2: variables identified via ML feature selection.

OR, odds ratio; CI, confidence interval; MET, metabolic equivalent of task; ML, machine learning, *p < 0.001.

**Table 3:** Complex sample logistic regression results (subjective stress level).

Variables			Subjective Stress level (ref: lower)			
			Model 1		Model 2	
			OR(p)	95% CI	OR(p)	95% CI
Literature review	Subjective health level	Good	1.00		1.00	
		General	2.01^*^	(2.00–2.03)	1.95^*^	(1.93–1.97)
		Bad	5.06^*^	(4.98–5.15)	4.48^*^	(4.41–4.56)
	Smoking	No	1.00			
		Yes	1.43^*^	(1.42–1.45)		
	Drinking	No	1.00			
		Yes	1.20^*^	(1.18–1.21)		
	Having	Yes	1.00			
	Breakfast	No	1.19^*^	(1.18–1.20)		
	Sleep	≥7 h	1.00		1.00	
		<7 h	1.62^*^	(1.60–1.63)	1.59^*^	(1.57–1.60)
	MET	<600	1.00		1.00	
		≥600	1.10^*^	(1.08–1.10)	1.08^*^	(1.07–1.09)
Feature selection	Unmet medical needs	No			1.00	
		Yes			1.97^*^	(1.94–2.00)
		Never needed			0.91^*^	(0.90–0.92)
	Chewing difficulty	No			1.00	
		General			1.37^*^	(1.35–1.39)
		Yes			1.56^*^	(1.53–1.59)
	Accident and addiction experience	No			1.00	
		1–2			1.36^*^	(1.33–1.38)
		More than 3			2.36^*^	(2.21–2.52)
Cox and Snell *R*^2^			0.075		0.079	
Nagelkerke *R*^2^			0.108		0.114	

Adjusted for age, sex, education, marriage, economic activity, and region.

Model 1: theoretical background variables identified in a literature review; Model 2: variables identified via ML feature selection.

OR, odds ratio; CI, confidence interval; MET, metabolic equivalent of task; ML, machine learning, *p < 0.001.

Regarding subjective stress levels, all variables had a significant impact (*p* < 0.001). Subjective health had the highest impact, whereby people with worse subjective health were more vulnerable to subjective stress. In the theoretical background variables, people who smoke or who sleep for less than 7 h at night were more likely to experience higher levels of subjective stress. Regarding the key factors, people experiencing unmet medical needs and chewing difficulty or more accident/addiction experiences tended to be more susceptible to subjective stress. Comparison of Models 1 and 2 for both dependent variables revealed that the odds ratio (OR) values of key factors were generally higher than those of theoretical background variables. Results of the complex sample logistic regression for all models are presented in Supplementary Tables 1, 2.

### 3.3 Key factors in the integrated model

**Figure 4:**
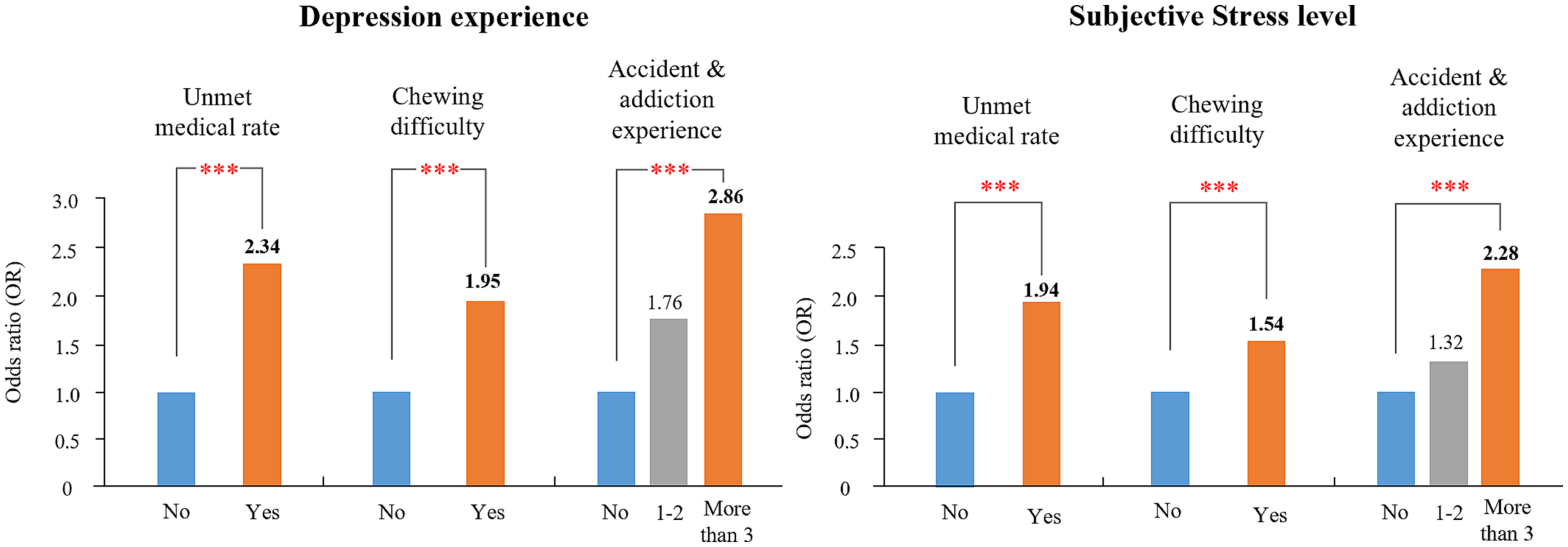
OR values of key factors in Model 3. ***P < 0.001.

We compared the impact of theoretical background variables and key factors on mental health by constructing an integrated model, Model 3. Figure 4 shows the OR values of the key factors associated with depression experience and subjective stress level. Both mental health variables showed a similar pattern, with the highest OR value for accidents/addiction, followed by unmet medical needs and chewing difficulty.

## 4 Discussion

We explored the factors influencing the mental health of young adults, considering changes in socioeconomic and cultural environments. We integrated a review of the literature with statistical analysis and ML based on the most up-to-date data available to reflect recent changes. We identified previously undiscussed variables affecting young adults’ mental health, such as unmet medical needs, chewing difficulty, and accident/addiction experiences; these significantly impacted depression and subjective stress.

Sex, subjective health level, sleep, and alcohol consumption are commonly related to depression and stress in young adults (34–37). Young adulthood is generally perceived as a healthy stage in the life cycle, with low use of medical services. However, in Korea (38) and elsewhere (39, 40) young adults are in fact more likely than older adult to experience unmet medical needs, whereby the availability of certain services is compromised by lengthy waiting times in hospitals or the lack of medical resources in certain residential areas (41). Recently, the emergence and development of various digital health technologies, such as mobile-health and AI-based health platforms, have increased expectations regarding healthcare services among young people, who engage extensively with social media (42). Arguably, therefore, young adults are more likely today to complain about their unmet medical needs arising from temporal constraints (43) or lack of satisfaction with medical services (44). Unmet medical needs are significantly correlated with high levels of stress and depression (38) and strongly associated with depression in young adults (45).

Young adulthood is associated with a high frequency of trauma and accidents (46), with a rate that is higher than those of other age groups (47, 48). Young adults’ use of digital devices has rapidly increased in recent years, and as the digital environment continues to change, the possibility of new threats, such as cyberbullying or cyberstalking, is also increasing (49). These can lead to symptoms of post-traumatic stress disorder (PTSD) and contribute to other mental health problems, such as depression, anxiety, and suicidal thoughts (50–52). Previous studies have also demonstrated that young adults who experience trauma are more likely to develop extensive mental health problems, including PTSD (53). PTSD is not a temporary condition but can degrade an individual’s daily life and social function in the long term. Therefore, early intervention to mediate traumatic experiences during young adulthood is crucial.

Furthermore, young adults often engage in substance use (54). Substance addiction has become a serious problem (55) as a result of recent trends such as the increasing use of e-cigarettes and synthetic drugs (56), and increased exposure to these substances via social media and online (57). Addiction to substances is associated with mental disorders in young adults and may even be a contributing factor (58). Use of multiple substances, in particular, may be an important predictor of increased depression. Recent studies have demonstrated that the concurrent use of both alcohol and cannabis may be associated with depressive symptoms in young adults (59). Stress in young adults is associated with addiction (60), and stressful life events can lead to substance abuse (61). Therefore, future research that considers the interaction between substance use, addiction, and mental health in young adults is warranted.

Young adults often have cavities and periodontal diseases stemming from childhood and may need to have several teeth extracted (62). According to the 2017 National Health Statistics, the prevalence of periodontal disease in young adults aged 19–29 years has shown a continuous annual increase (63). Research into Korean adults’ oral health also indicates a higher proportion of teeth requiring treatment or extraction with a decrease in age (64). Moreover, young adults are increasingly seeking orthodontic treatment to address malocclusion or aesthetic concerns (65). Cavities, periodontal disease, tooth loss, and malocclusion can all affect oral health and cause chewing difficulties. Chewing difficulties are also associated with emotions and can diminish individuals’ willingness to participate in various activities (66), leading to depression and stress (67, 68). Notably, individuals experiencing chewing problems have been shown to exhibit a progressively higher risk of depressive symptoms, indicating that such difficulties may have a stronger association with moderate to severe depression than with mild symptoms (69). Given that most oral health projects are focused on the older adult or children, policies aimed specifically at improving young adults’ oral health are required.

In this study, we proposed a hybrid ML approach to address the limitations of other statistical analysis methods. Traditional methods identify important variables for analyzing risk factors of specific diseases based on medical knowledge, theoretical background, and literature reviews. However, the process of variable selection based on theoretical grounds cannot wholly exclude subjectivity (70). As an alternative, ML-based feature selection methods have been suggested (71) and used in several recent studies (72, 73). ML-based methods efficiently extract the most relevant features when analyzing various variables from a large dataset (74) but tend to be more complex and less interpretable than more traditional approaches (75). While they are useful for evaluating variables that are not predicted by traditional modeling, their ability to show the direction of association is limited (76). Using a combination of both methods can facilitate the exploration of influencing factors and effectively analyze their impact. Russel et al. (77) found that feature selection complemented logistic regression and identified new variables, demonstrating its value when used with traditional statistical methods. In this study, we compared models based on a literature review and ML feature selection to identify factors not previously considered in the literature.

### 4.1 Limitations

This study is significant in that it applied exploratory analysis to a nationwide representative sample to identify the factors affecting young adults’ mental health across a wide range of categories. Nevertheless, there are limitations to mention. First, as a cross-sectional study that used data from the CHS, it is difficult to identify the causal relationship between the selected variables and mental health. Second, self-reported mental health status and other variables (health behaviors, etc.) are susceptible to recall and social biases and thus may not provide accurate information. Finally, the findings should be interpreted with caution, as the data are derived from a specific sociocultural setting, limiting their generalizability to other populations with different cultural backgrounds.

## 5 Conclusion

We applied a hybrid ML/traditional statistics methodology to identify and explore the factors influencing young adults’ mental health in light of recent environmental and lifestyle changes. Unmet medical needs, chewing difficulties, and the number of accidents and addiction experiences were newly derived key factors. We confirmed the effects of these factors that have previously largely been overlooked. This work emphasizes the need to establish policies aimed at managing young adults’ mental health by continuously monitoring the influencing factors in a rapidly changing environment.

## Data Availability

Publicly available datasets were analyzed in this study. This data can be found at: Korea Disease Control and Prevention Agency.
