# Supplementary material for: Change in lifestyle and mental health in young adults: an exploratory study with hybrid machine learning
Source: Front Public Health. 2025 Jun 4;13:1562280. doi: 10.3389/fpubh.2025.1562280 (PMC12173860; doi:10.3389/fpubh.2025.1562280)
Supplement: Supplementary file 1 [file Table_1.docx]

Supplementary Material

# Supplementary Table

Supplementary Table 1 Complex sample logistic regression results of All Models (Depression experience)

| Variables | | | Depression experience (ref: No) | | | | | | | | |
| --- | --- | --- | --- | --- | --- | --- | --- | --- | --- | --- | --- |
|  |  |  | Model1 | | | Model2 | | | Model3 | | |
|  |  |  | OR(p) | (95% CI) | | OR(p) | (95% CI) | | OR(p) | (95% CI) | |
| Socio-demo graphic | Gender | male | 1.00 |  |  | 1.00 |  |  | 1.00 |  |  |
|  |  | female | 2.15^*^ | (2.11- | 2.19) | 1.74^*^ | (1.71- | 1.76) | 2.07^*^ | (2.03- | 2.11) |
|  | Age | 19-29 | 1.00 |  |  | 1.00 |  |  | 1.00 |  |  |
|  |  | 30-34 | 1.05 | (1.03- | 1.06) | 1.05^*^ | (1.03- | 1.07) | 1.05^*^ | (1.04- | 1.07) |
|  | Education | Middle school and below | 1.00 |  |  | 1.00 |  |  | 1.00 |  |  |
|  |  | High school | 0.71^*^ | (0.66- | 0.77) | 0.77^*^ | (0.71- | 0.83) | 0.78^*^ | (0.71- | 0.84) |
|  |  | University or higher | 0.53^*^ | (0.49- | 0.58) | 0.57^*^ | (0.52- | 0.61) | 0.60^*^ | (0.55- | 0.65) |
|  | Marriage | married | 1.00 |  |  | 1.00 |  |  | 1.00 |  |  |
|  |  | bereavement, divorce | 1.85^*^ | (1.75- | 1.95) | 1.88^*^ | (1.78- | 1.99) | 1.83^*^ | (1.73- | 1.93) |
|  |  | single | 1.06^*^ | (1.03- | 1.09) | 1.10^*^ | (1.08- | 1.13) | 1.10^*^ | (1.08- | 1.13) |
|  | Economic activity  for the last week | Yes | 1.00 |  |  | 1.00 |  |  | 1.00 |  |  |
|  |  | No | 1.16^*^ | (1.14- | 1.17) | 1.10^*^ | (1.08- | 1.11) | 1.18^*^ | (1.16- | 1.20) |
|  | Household  income | High | 1.00 |  |  | 1.00 |  |  | 1.00 |  |  |
|  |  | Middle | 1.15^*^ | (1.13- | 1.17) | 1.15^*^ | (1.13- | 1.17) | 1.13^*^ | (1.11- | 1.15) |
|  |  | Low | 1.58^*^ | (1.55- | 1.61) | 1.57^*^ | (1.54- | 1.60) | 1.53^*^ | (1.50- | 1.56) |
|  | Region | metropolitan | 1.00 |  |  | 1.00 |  |  | 1.00 |  |  |
|  |  | non-metropolitan | 0.85^*^ | (0.83- | 0.87) | 0.81^*^ | (0.79- | 0.83) | 0.83^*^ | (0.80- | 0.85) |
| Theoretical  background | Subjective  health level | Good | 1.00 |  |  | 1.00 |  |  | 1.00 |  |  |
|  |  | General | 2.11^*^ | (2.07- | 2.14) | 1.96^*^ | (1.93- | 1.99) | 1.92^*^ | (1.89- | 1.95) |
|  |  | Bad | 6.22^*^ | (6.01- | 6.36) | 5.00^*^ | (4.88- | 5.12) | 4.85^*^ | (4.74- | 4.97) |
|  | Smoking | No | 1.00 |  |  |  |  |  | 1.00 |  |  |
|  |  | Yes | 1.71^*^ | (1.68- | 1.75) |  |  |  | 1.62^*^ | (1.59- | 1.66) |
|  | Drinking | No | 1.00 |  |  |  |  |  | 1.00 |  |  |
|  |  | Yes | 1.19^*^ | (1.16- | 1.22) |  |  |  | 1.16^*^ | (1.13- | 1.19) |
|  | Having Breakfast | Yes | 1.00 |  |  |  |  |  | 1.00 |  |  |
|  |  | No | 0.98^*^ | (0.97- | 0.99) |  |  |  | 0.97^*^ | (0.96- | 0.99) |
|  | Sleep | ≥ 7 hours | 1.00 |  |  |  |  |  | 1.00 |  |  |
|  |  | ＜ 7 hours | 1.36^*^ | (1.34- | 1.38) |  |  |  | 1.29^*^ | (1.27- | 1.31) |
|  | MET | ＜ 600 | 1.00 |  |  |  |  |  | 1.00 |  |  |
|  |  | ≥ 600 | 1.26^*^ | (1.24- | 1.28) |  |  |  | 1.25^*^ | (1.23- | 1.27) |
| Key  factor | Unmet  medical rate | No |  |  |  | 1.00 |  |  | 1.00 |  |  |
|  |  | Yes |  |  |  | 2.43^*^ | (2.37- | 2.50) | 2.34^*^ | (2.28- | 2.41) |
|  |  | Never needed |  |  |  | 0.89^*^ | (0.87- | 0.91) | 0.89^*^ | (0.87- | 0.91) |
|  | Chewing  difficulty | No |  |  |  | 1.00 |  |  | 1.00 |  |  |
|  |  | General |  |  |  | 1.55^*^ | (1.51- | 1.58) | 1.51^*^ | (1.47- | 1.55) |
|  |  | Yes |  |  |  | 2.01^*^ | (1.95- | 2.06) | 1.95^*^ | (1.90- | 2.01) |
|  | Accident and  addiction  experience | No |  |  |  | 1.00 |  |  | 1.00 |  |  |
|  |  | 1~2 |  |  |  | 1.85^*^ | (1.79- | 1.90) | 1.76^*^ | (1.71- | 1.82) |
|  |  | more than 3 |  |  |  | 3.15^*^ | (2.79- | 3.56) | 2.86^*^ | (2.56- | 3.19) |
| Cox & Snell R^2^ | | | 0.036 | | | 0.041 | | | 0.044 | | |
| Nagelkerke R^2^ | | | 0.098 | | | 0.110 | | | 0.119 | | |

Model 1: theoretical background variables identified in a literature review

Model 2: variables identified via ML feature selection.

Model 3 : Integration of variables in Model 1 and Model 2

OR: odds ratio, CI: confidence interval, MET: metabolic equivalent of task, ML: machine learning, *p < 0.001

Supplementary Table 2 Complex sample logistic regression results of All Models (Subjective stress level)

| Variables | | | Subjective Stress level(ref: Lower) | | | | | | | | |
| --- | --- | --- | --- | --- | --- | --- | --- | --- | --- | --- | --- |
|  |  |  | Model1 | | | Model2 | | | Model3 | | |
|  |  |  | OR(p) | (95% CI) | | OR(p) | (95% CI) | | OR(p) | (95% CI) | |
| Socio-demo graphic | Gender | male | 1.00 |  |  | 1.00 |  |  | 1.00 |  |  |
|  |  | female | 1.53^*^ | (1.52- | 1.54) | 1.36^*^ | (1.35- | 1.37) | 1.49^*^ | (1.48- | 1.51) |
|  | Age | 19-29 | 1.00 |  |  | 1.00 |  |  | 1.00 |  |  |
|  |  | 30-34 | 1.14^*^ | (1.13- | 1.15) | 1.13^*^ | (1.12- | 1.14) | 1.14^*^ | (1.13- | 1.15) |
|  | Education | Middle school and below | 1.00 |  |  | 1.00 |  |  | 1.00 |  |  |
|  |  | High school | 0.92^*^ | (0.88- | 0.96) | 0.96^*^ | (0.92- | 1.01) | 0.97^*^ | (0.93- | 1.01) |
|  |  | University or higher | 0.83^*^ | (0.79- | 0.86) | 0.84^*^ | (0.81- | 0.88) | 0.89^*^ | (0.85- | 0.93) |
|  | Marriage | married | 1.00 |  |  | 1.00 |  |  | 1.00 |  |  |
|  |  | bereavement, divorce | 1.22^*^ | (1.18- | 1.26) | 1.23^*^ | (1.19- | 1.28) | 1.21^*^ | (1.17- | 1.25) |
|  |  | single | 0.91^*^ | (0.90- | 0.92) | 0.93^*^ | (0.92- | 0.94) | 0.93^*^ | (0.92- | 0.94) |
|  | Economic activity  for the last week | Yes | 1.00 |  |  | 1.00 |  |  | 1.00 |  |  |
|  |  | No | 0.76^*^ | (0.75- | 0.77) | 0.73^*^ | (0.73- | 0.74) | 0.77^*^ | (0.76- | 0.77) |
|  | Household  income | High | 1.00 |  |  | 1.00 |  |  | 1.00 |  |  |
|  |  | Middle | 1.07^*^ | (1.06- | 1.08) | 1.08^*^ | (1.06- | 1.09) | 1.06^*^ | (1.05- | 1.08) |
|  |  | Low | 1.07^*^ | (1.06- | 1.22) | 1.21^*^ | (1.20- | 1.22) | 1.18^*^ | (1.17- | 1.20) |
|  | Region | metropolitan | 1.00 |  |  | 1.00 |  |  | 1.00 |  |  |
|  |  | non-metropolitan | 0.91^*^ | (0.90- | 0.92) | 0.90^*^ | (0.88- | 0.91) | 0.89^*^ | (0.88- | 0.90) |
| Theoretical  background | Subjective  health level | Good | 1.00 |  |  | 1.00 |  |  | 1.00 |  |  |
|  |  | General | 2.01^*^ | (2.00- | 2.03) | 1.95^*^ | (1.93- | 1.97) | 1.92^*^ | (1.90- | 1.93) |
|  |  | Bad | 5.06^*^ | (4.98- | 5.15) | 4.48^*^ | (4.41- | 4.56) | 4.41^*^ | (4.33- | 4.48) |
|  | Smoking | No | 1.00 |  |  |  |  |  | 1.00 |  |  |
|  |  | Yes | 1.43^*^ | (1.42- | 1.45) |  |  |  | 1.40^*^ | (1.38- | 1.41) |
|  | Drinking | No | 1.00 |  |  |  |  |  | 1.00 |  |  |
|  |  | Yes | 1.20^*^ | (1.18- | 1.21) |  |  |  | 1.17^*^ | (1.16- | 1.19) |
|  | Having Breakfast | Yes | 1.00 |  |  |  |  |  | 1.00 |  |  |
|  |  | No | 1.19^*^ | (1.18- | 1.20) |  |  |  | 1.18^*^ | (1.18- | 1.19) |
|  | Sleep | ≥ 7 hours | 1.00 |  |  | 1.00 |  |  | 1.00 |  |  |
|  |  | ＜ 7 hours | 1.62^*^ | (1.60- | 1.63) | 1.59^*^ | (1.57- | 1.60) | 1.58^*^ | (1.57- | 1.60) |
|  | MET | ＜ 600 | 1.00 |  |  | 1.00 |  |  | 1.00 |  |  |
|  |  | ≥ 600 | 1.10^*^ | (1.08- | 1.10) | 1.08^*^ | (1.07- | 1.09) | 1.08^*^ | (1.07- | 1.09) |
| Key  factor | Unmet  medical rate | No |  |  |  | 1.00 |  |  | 1.00 |  |  |
|  |  | Yes |  |  |  | 1.97^*^ | (1.94- | 2.00) | 1.94^*^ | (1.91- | 1.97) |
|  |  | Never needed |  |  |  | 0.91^*^ | (0.90- | 0.92) | 0.90^*^ | (0.89- | 0.91) |
|  | Chewing  difficulty | No |  |  |  | 1.00 |  |  | 1.00 |  |  |
|  |  | General |  |  |  | 1.37^*^ | (1.35- | 1.39) | 1.36^*^ | (1.34- | 1.37) |
|  |  | Yes |  |  |  | 1.56^*^ | (1.53- | 1.59) | 1.54^*^ | (1.51- | 1.57) |
|  | Accident and  addiction  experience | No |  |  |  | 1.00 |  |  | 1.00 |  |  |
|  |  | 1~2 |  |  |  | 1.36^*^ | (1.33- | 1.38) | 1.32^*^ | (1.30- | 1.35) |
|  |  | more than 3 |  |  |  | 2.36^*^ | (2.21- | 2.52) | 2.28^*^ | (2.14- | 2.43) |
| Cox & Snell R^2^ | | | 0.075 | | | 0.079 | | | 0.083 | | |
| Nagelkerke R^2^ | | | 0.108 | | | 0.114 | | | 0.120 | | |

Model 1: theoretical background variables identified in a literature review

Model 2: variables identified via ML feature selection.

Model 3 : Integration of variables in Model 1 and Model 2

OR: odds ratio, CI: confidence interval, MET: metabolic equivalent of task, ML: machine learning, *p < 0.001
